# Exposure to sucrose during periods of withdrawal does not reduce cocaine-seeking behavior in rats

**DOI:** 10.1038/srep23272

**Published:** 2016-03-21

**Authors:** Céline Nicolas, Claire Lafay-Chebassier, Marcello Solinas

**Affiliations:** 1 INSERM, U1084, F-86022 Poitiers, France; 2 Université de Poitiers, U1084, F-86022 Poitiers, France; 3 Department of Clinical Pharmacology, Poitiers University Hospital, F-86021 Poitiers-France

## Abstract

Concomitant access to drugs of abuse and alternative rewards such as sucrose has been shown to decrease addiction-related behaviors in animals. Here we investigated whether access to sucrose during abstinence in contexts that are temporally and physically distinct from drug-related contexts could reduce subsequent drug seeking. In addition, we investigated whether a history of cocaine self-administration would alter the rewarding effects of sucrose. Rats self-administered cocaine for ten sessions, while yoked-saline rats received only saline injections, and then we subjected them to a 30-day withdrawal period during which they had access to water and sucrose continuously or intermittently according to a schedule that induces binge-drinking behavior. At the end of the withdrawal period, rats were tested for cocaine seeking behavior during a single 6 h session. We found that exposure to cocaine increased sucrose consumption only when rats had intermittent access to sucrose, but exposure to sucrose did not alter drug seeking regardless of the schedule of access. These results suggest that exposure to cocaine cross-sensitizes to the rewarding effects of sucrose, but exposure to sucrose during abstinence, temporally and physically distinct from drug-related environments, does not to reduce drug seeking.

Exposure to environmental enrichment (EE) during periods of withdrawal from drugs has been shown to decrease drug seeking in animal models of relapse^1^,^2^,^3^,^4^,^5^. EE is a combination of physical, cognitive and social stimulation^6^, which are believed to synergize to produce beneficial effects in models of several pathologies^7^. One explanation for the positive effects of EE is that EE could act as an alternative reward^8^. As a matter of fact, social interactions^9^,^10^,^11^, novelty^12^,^13^ and voluntary physical activity^14^ that are part of EE, are all rewarding in rodents. In humans, alternative reinforcers, mostly provided in the form of monetary vouchers, significantly decrease relapse to several drugs of abuse^15^. In preclinical models, it has also been clearly demonstrated that when animals have access to non-drug reinforcers during self-administration sessions, drug taking and preference are significantly reduced^16^,^17^,^18^,^19^. Furthermore, when animals are forced to choose between a sweet solution and drugs of abuse in an operant cage, they mostly prefer sweet solutions and ignore the drugs^20^ and, even after long exposure to a highly addictive drug such as heroin, choice for drug does not surpass choice for saccharin solutions^21^. However, there are major differences between these studies and those showing anti-craving effects of EE. In fact, EE is provided as a housing condition during most of the day, for several days, outside self-administration cages^1^,^2^,^3^,^4^,^5^, whereas, in most studies, alternative reinforcement is given inside the self-administration cage for a limited time of the day^17^,^20^. Therefore, we designed this experiment to test the hypothesis that providing an alternative reinforcer such a sweet taste in a fashion similar to EE, during periods of withdrawal, would decrease cocaine seeking in an animal model of relapse^22^.

Sucrose and sweet tastes activate the reward system^23^,^24^ and increase dopamine levels in nucleus accumbens^25^. Importantly, chronic exposure to sucrose produces neuroadaptations in the amydgala that lead to a reduction of the neuroendocrine, cardiovascular, and behavioral responses to stress in rats^26^, suggesting that, similar to EE, chronic sucrose may have an anti-stress effect. The behavioral and neurobiological consequences of exposure to sweet solutions appear to depend on the schedule of administration, with intermittent access to sweet solutions producing sensitization of the reward circuit. For example, Hoebel and collaborators have shown that intermittent exposure to sucrose can produce effects similar to those of drugs of abuse, such as modifications in brain reward circuits and addiction-like behaviors^27^,^28^. Therefore, the main aim of this study was to test the ability of different schedules of sucrose access (a continuous access more associated with tolerance and intermittent access associated with sensitization of the reward pathway and binge-like behavior) to alter cocaine seeking.

Importantly, the ability of sucrose to act as a natural reward has been frequently used as a measure of hedonia/anhedonia and therefore, it has been used as a measure of depression^29^,^30^. Drug addiction has been suggested to result in the dysregulation of reward function and in depression-like states. Indeed, measures of reduced activity of the reward system such as intracranial self-stimulation have shown that after short, but not long, periods of withdrawal the reward system is hypo-functional^31^. Conversely, exposure to drugs may sensitize animals to sucrose rewarding effects leading to excessive consumption of sucrose when sucrose is available intermittently^32^. Therefore, a secondary aim of this study was to monitor consumption of sweet solutions during different phases of abstinence to investigate whether withdrawal from cocaine is associated with anhedonia or sensitization of sucrose, as well as the evolution of such a phenomenon over the course of the withdrawal period.

In this study, first, rats were allowed to self-administer cocaine and were then exposed to water or sucrose for 30 days of forced abstinence (see Fig. 1, for schematic representation of the experimental design). One group had access to two bottles of water (WAT), one group had a continuous access to one bottle of water and one bottle of sucrose (ContS) and one group had an intermittent access to water or sucrose (IntS) according to the protocol developed by Hoebel and collaborators^27^,^28^. After 30 days of withdrawal, rats were tested for cocaine seeking during a 6 h session in an animal model of relapse^22^.

## Results

### Experiment 1

#### Cocaine self-administration training

Given our experimental design, cocaine self-administration did not differ among rats that were later assigned to WAT or ContS conditions in terms of average (±SEM) number of injections (110 ± 13 and 113 ± 16), number of active nose-pokes (160 ± 31 and 167 ± 27) or inactive nose-pokes (37 ± 16 and 32 ± 8) (data not shown graphically).

#### Liquid consumption behavior

In rats that had access to water only (WAT) during a 30-day period of withdrawal from cocaine self-administration, water intake was stable (74.9 ± 3.9 g/48 h) during the entire withdrawal period and did not differ between cocaine and saline rats (Fig. 2A,B).

Rats that had continuous access to sucrose (ContS) during a 30-day period of withdrawal avidly consumed sucrose and completely ignored water (Fig. 2C,D). In fact, they drank 203.9 ± 18.6 g/48 h of sucrose and only 3.7 ± 1.4 g/48 h of water (Fig. 2C,D) and they showed 98.1 ± 0.6% of preference for sucrose (Fig. 2E,F). During the first four days, sucrose consumption appeared slightly lower in cocaine-exposed rats (151.4 ± 16.8 g/48 h) compared to saline-exposed rats (180.2 ± 25 g/48 h), but this difference did not reach statistical significance. Subsequently, sucrose consumption increased similarly in the two groups and reached an average of 219.2 ± 18.6 g/48 h in both groups during the last six days (Fig. 2C). For sucrose consumption, statistical analysis revealed no significant effect of drug, but there was a significant effect of time [F(15, 225) = 6.5, p < 0.0001] and significant drug X time interaction [F(15, 225) = 2.2, p < 0.01]. For water consumption or sucrose preference, statistical analysis revealed no significant effect of drug, time or drug X time interaction.

Total liquid consumption during the withdrawal period was much higher (276%) in ContS compared to WAT rats (Fig. S1), which further demonstrates the strong rewarding effect of sucrose. Statistical analysis revealed a significant effect of liquid [F(1, 31) = 67.45, p < 0.0001], but no significant effect of drug or liquid X drug interaction.

#### Cocaine-seeking behavior

Cocaine seeking was investigated after 30-day period of withdrawal during a single 6 h session divided in six 1 h intervals (Fig. 3A,B). As expected, the number of active nose-pokes was higher in rats with a previous history of cocaine compared to saline-yoked rats. Continuous access to sucrose over the withdrawal period did not modify levels of response in cocaine groups compared to rats with an access to water only. Statistical analysis revealed a significant effect of drug [F(1, 41) = 32.30, p < 0.0001], but no effect of liquid or liquid X drug interaction.

### Experiment 2

#### Cocaine self-administration training

Again, cocaine self-administration did not differ among rats that were later assigned to WAT or IntS conditions in terms of average number (±SEM) of injections (103 ± 6 and 101 ± 10), number of active nose-pokes (176 ± 45 and 167 ± 55) and inactive nose-pokes (29 ± 8 and 27 ± 9) (data not shown graphically).

#### Liquid consumption behavior

In rats that had access to water only (WAT) during a 30-day period of withdrawal from cocaine self-administration, water intake was stable at 11.3 ± 0.7 g/12 h during the entire withdrawal period and did not differ between cocaine and saline rats (Fig. 4A,B).

Rats that had intermittent access to sucrose (IntS) during a 30-day period of withdrawal, avidly consumed sucrose whenever sucrose solutions were available and increased sucrose consumption over time (Fig. 4C,D). Interestingly, cocaine-exposed and yoked-control rats had similar intake of sucrose during the first days of withdrawal and exposure to sucrose but after about 5 days, cocaine-exposed rats started to show consistently a higher intake of sucrose compared to yoked-control rats (79.0 ± 6.9 vs 60.1 ± 7.2 g/12 h) (Fig. 4C,D). Statistical analysis revealed a significant effect of drug [F(1, 16) = 4.87, p < 0.05], a significant effect of time [F(28, 448) = 14.41, p < 0.0001)] and a significant drug X time interaction [F(28, 448) = 1.89, p < 0.01]. Under conditions of intermittent access, sucrose preference was 74.1 ± 5.7% and 69.4 ± 6.3% on the first day and 96 ± 1.6% and 90.6 ± 4.1% for the remainder of the study in cocaine-exposed and yoked-control rats, respectively (Fig. 4E,F). However this difference did not reach significance.

During the 12 h period in which both sucrose and water were available, total liquid consumption was much higher in IntS compared to WAT rats and this effect was significantly stronger in cocaine-exposed rats (702% increase) compared to yoked-saline controls (547%) (student T-Test, p < 0.01) (Fig. S2). Statistical analysis revealed a significant effect of liquid [F(1, 32) = 297.52, p < 0.0001], a trend for a significant effect of drug (p = 0.054) and a significant liquid X drug interaction [F(1, 32) = 4.63, p < 0.05].

#### Cocaine-seeking behavior

Cocaine seeking was investigated after 30-day period of withdrawal during a single 6 h session divided in six 1 h intervals (Fig. 5A,B). As expected, the number of active nose-pokes was higher in rats with a previous history of cocaine compared to saline-yoked rats. Intermittent access to sucrose over the withdrawal period did not modify levels of response in cocaine groups compared to rats with access to water only. Statistical analysis revealed a significant effect of drug [F(1, 41) = 32.30, p < 0.0001], but no effect of liquid or liquid X drug interaction.

## Discussion

In this study, our primary aim was to investigate whether a chronic exposure to sucrose would reduce cocaine seeking in rats in a relapse model and our secondary aim was to investigate whether a history of cocaine self-administration would alter the rewarding effect of sucrose. We found that rats avidly consumed sucrose and that previous exposure to cocaine increased motivation for sucrose, but only when sucrose was available intermittently according to a protocol that induces binge-drinking behavior. However, notwithstanding the strong rewarding effects of sucrose, exposure to sucrose did not alter drug-seeking behavior. This suggests that chronic exposure to an alternative reward outside of the self-administration setting does not decrease cocaine craving. Moreover, this suggests that the anti-craving effects of EE cannot be solely attributed to alternative rewards.

Exposure to drugs is known to alter activity of the reward pathway^33^,^34^. These alterations may lead to either decreases or increases in the reactivity to natural rewards. For example, addiction may be associated with anhedonia, i.e. a reduction in the pleasure produced by rewarding stimuli^34^. While the theoretical basis for this hypothesis is solid and clinical evidence appears to support it^35^, few preclinical studies have been able to detect decreased reactivity to rewards after psychostimulant administration and in most cases these effects were limited to early stages of withdrawal. A seminal paper by Ahmed *et al.* demonstrated that escalation of cocaine self-administration increases the threshold for intracranial self-stimulation i.e. decreases reward sensitivity^31^. Two recent studies found decreases in sucrose preference or reduced learning of behavioral task motivated by sucrose after sensitization to cocaine^36^,^37^. Finally, a more recent study using a choice procedure showed that after prolonged heroin^21^, but not cocaine exposure^20^, rats decrease their choice for sweet solutions. On the other hand, exposure to drugs has also been shown to sensitize the reward pathway and this effect has been hypothesized to play a major role in the abnormal salience attribution found in addiction^38^,^39^. An interesting feature of our experimental design is that while sucrose exposure was used as an independent variable, sucrose intake could be also used as a dependent variable to determine whether cocaine self-administration alters the reactivity to natural rewards. Our results show that the consequences of exposure to cocaine on sucrose consumption depend critically on the protocol of access to sucrose. Thus, under conditions of continuous access, sucrose consumption was not significantly altered by cocaine exposure (although a tendency for a decrease sucrose consumption was observed during the first days), whereas under conditions of intermittent access, sucrose consumption was significantly increased. Therefore, it appears that a history of cocaine administration does not reduce but rather cross-sensitize to the rewarding effects of sucrose but only when rats have limited, intermittent access to sucrose. These results are in agreement with a previous paper showing that exposure to amphetamine increases sucrose consumption under conditions of intermittent access^32^. Therefore, these results suggest that abstinence from cocaine self-administration could facilitate the development of other dysregulated and excessive behaviors towards non-drug rewards.

The main aim of this study was to draw a parallel between exposure to sucrose and exposure to EE. In fact, several recent studies have shown that exposure to EE during periods of withdrawal decreases drug-seeking behavior^1^,^2^,^3^,^4^,^5^,^40^ and it could be argued that EE produced its anti-craving effects by acting as an alternative reward^8^. Sucrose has powerful reinforcing effects in rodents^41^ and similarly to exposure to EE^8^, exposure to sucrose has been to show to have powerful anti-stress effects^26^. Finally, a vast literature supports the positive effects of alternative rewards on addiction^42^,^43^. However, the experimental designs that have been mostly used in those studies are fundamentally different from exposure to EE. In fact, in preclinical setting, alternative rewards are usually given within the self-administration setting, either as an option or as a choice^42^,^43^. These procedures and especially choice procedures may be seen as analogs of contingency-management therapy in humans, in which for example, money vouchers are given to promote abstinence in addicts provided that they forgo drug use^44^,^45^. On the other hand, EE rats live in complex environments and thus are provide natural rewards outside of the self-administration setting^1^,^2^,^3^,^4^,^5^,^40^. To mimic exposure to EE, rats in the current study had access to sucrose only in their housing cages during periods of abstinence and sucrose was not available in the self-administration cage. However, contrary to our working hypothesis, we found that although rats found sweet solutions highly rewarding, exposure to sucrose did not alter cocaine-seeking behavior. This suggests that exposure to an alternative reward per se during periods of withdrawal is not sufficient to decrease drug seeking behavior. Therefore, whereas alternative rewards produce striking decreases in drug-taking and drug-seeking behaviors when they compete for drug reinforcement^42^,^43^, alternative rewards are not effective in reducing relapse when presented non-contingently outside of the self-administration setting. If these results are translated to humans, they appear to suggest that whereas contingency management therapy is effective, the simple presence of an alternative reward in the life of abstinent individuals may not be sufficient to drive the brain adaptations that promote abstinence.

In this study, in order to draw a parallel between sucrose exposure and EE, we used a protocol of sucrose exposure that maximized voluntary sucrose consumption that resembled as much as possible previous studies with EE^1^,^2^,^3^,^4^,^5^,^40^. A possible limitation of the present study is that we used only one concentration of sucrose (10%) and two protocols of exposure to sucrose (continuous and intermittent) and therefore, we cannot rule out that, using different conditions of sucrose access, we would have obtained different results. For example, it could be speculated that by using different concentrations of sucrose, we could have revealed significant cocaine-induced anhedonia-like effects. Conversely, it is possible that using protocols of access to sucrose that involve some kind of novelty, we could have obtained synergistic effects of non-drug reward and novelty, and consequently a reduction in cocaine-seeking behavior. For example, a previous study found that a single, short exposure to a sweet taste immediately prior to a cocaine seeking test session, significantly reduces cocaine seeking^46^. Future studies may be needed to obtain a more complete and definitive picture of the reciprocal interactions between cocaine- and sucrose-related behaviors.

In conclusion, in this study we found that exposure to cocaine leads to a persistent increase in the motivational effects of sucrose under conditions of intermittent exposure leading to exacerbation of binge-drinking behavior. Therefore, cocaine appears to sensitize the reward system leading to a higher risk to develop excessive behaviors towards non-drug rewards. Notwithstanding these powerful rewarding effects, sucrose was not able to reduce cocaine seeking behavior, suggesting that exposure to non-drug rewards alone is not sufficient to prevent craving for the drug. These results suggest that aspects of EE other than alternative reward such as physical exercise, social contact, novelty, etc may be more important than alternative reinforcement in producing EE’s effects or the combination of alternative reward with several enriching factors may have synergistic effects that are greater than either of these factors taken alone.

## Methods

### Subjects and housing conditions

Adult (8 weeks of age) male Sprague Dawley rats (Janvier, France), experimentally naïve at the beginning of the study, were housed in a temperature and humidity controlled environment, and maintained on a 12-h light/dark cycle (light on at 7 AM). All experiments were conducted in accordance to European Union directives (2010/63/EU) for the care of laboratory animals and all experimental protocols were approved by the local ethics committee (COMETHEA). Upon arrival, rats were housed two per cage for about 1 week before intra-jugular vein catheterization surgery. After surgery, rats were housed individually for the rest of the study in order to quantify individual drinking behavior.

### General experimental design

**Figure 1:**
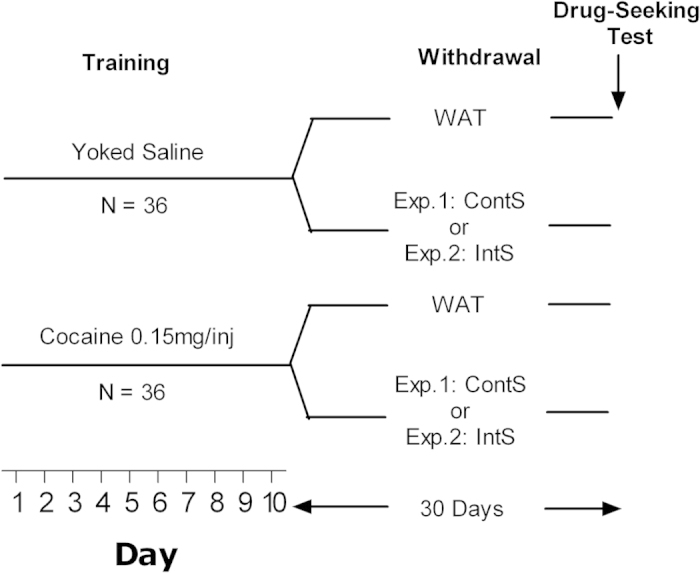
Schematic representation of the experimental design used. After 10 days of cocaine or yoked saline self-administration, rats were subjected to 30-day withdrawal during which they had access to water only (WAT), to water and sucrose (10% w/v) continuously (experiment 1) or intermittently (experiment 2). At the end of withdrawal period, rats were tested for cocaine seeking during 6 h session.

**Figure 2:**
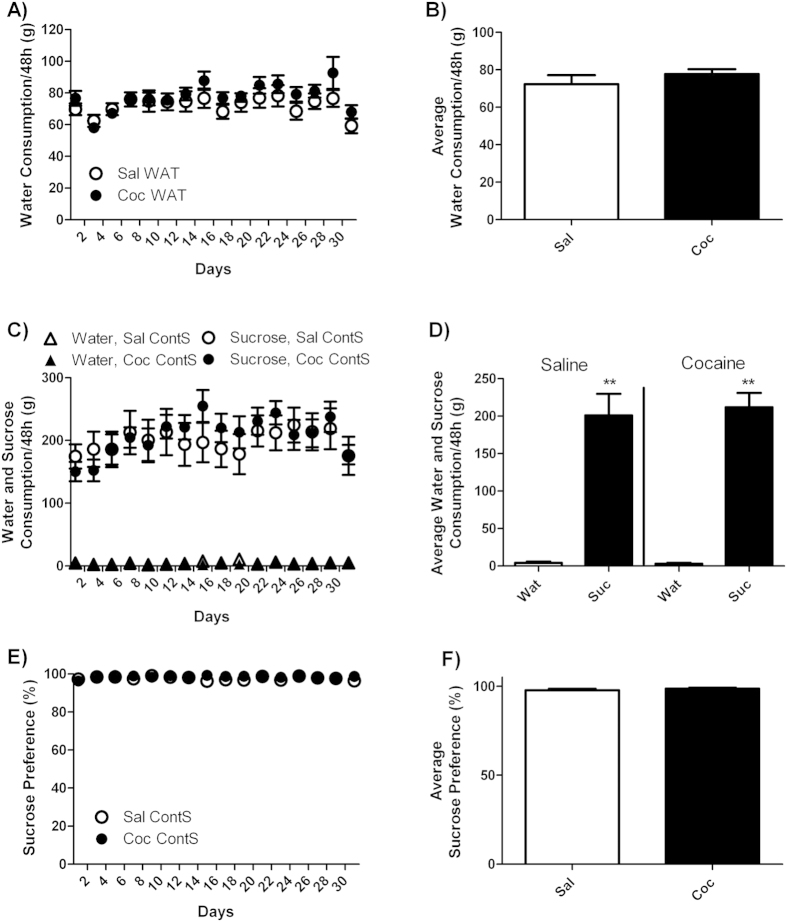
Water (Wat) and sucrose (Suc) consumption in rats with continuous access to sucrose. (**A**) Time-course of water consumption measured every 48 h and (**B**) average water consumption during withdrawal period for yoked-saline (Sal WAT) and cocaine-exposed (Coc WAT) groups in animals that had access to water only. (**C**) Time-course of sucrose and water consumption and (**E**) sucrose preference measured every 48 h and (**D**) average sucrose and water consumption and (**F**) average sucrose preference during the withdrawal period for yoked-saline (Sal ContS) and cocaine-exposed (Coc ContS) groups in animals that had continuous access to sucrose. Sucrose preference was expressed as percentage of total intake. Two-Way ANOVA followed by Student-Neuman-Keuls post-hoc test, **p < 0.01 Suc compared to WAT.

**Figure 3:**
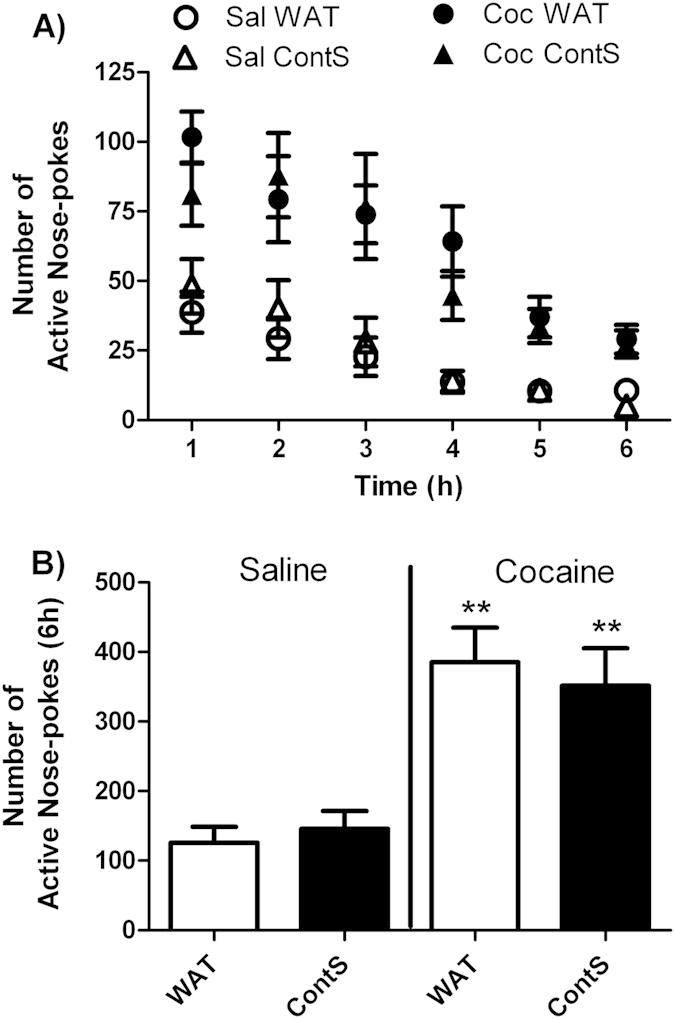
Lack of effect of continuous exposure to sucrose during withdrawal period on cocaine seeking behavior. Time course (**A**) and total (**B**) cocaine seeking measured by the number of active nose-pokes during 6 h-session in yoked-saline and cocaine-exposed groups and access to water (WAT) or continuous sucrose (ContS) during a 30-day withdrawal period. Two-Way ANOVA followed by Student-Neuman-Keuls post-hoc test, **p < 0.01 cocaine compared to respective saline yoked controls.

**Figure 4:**
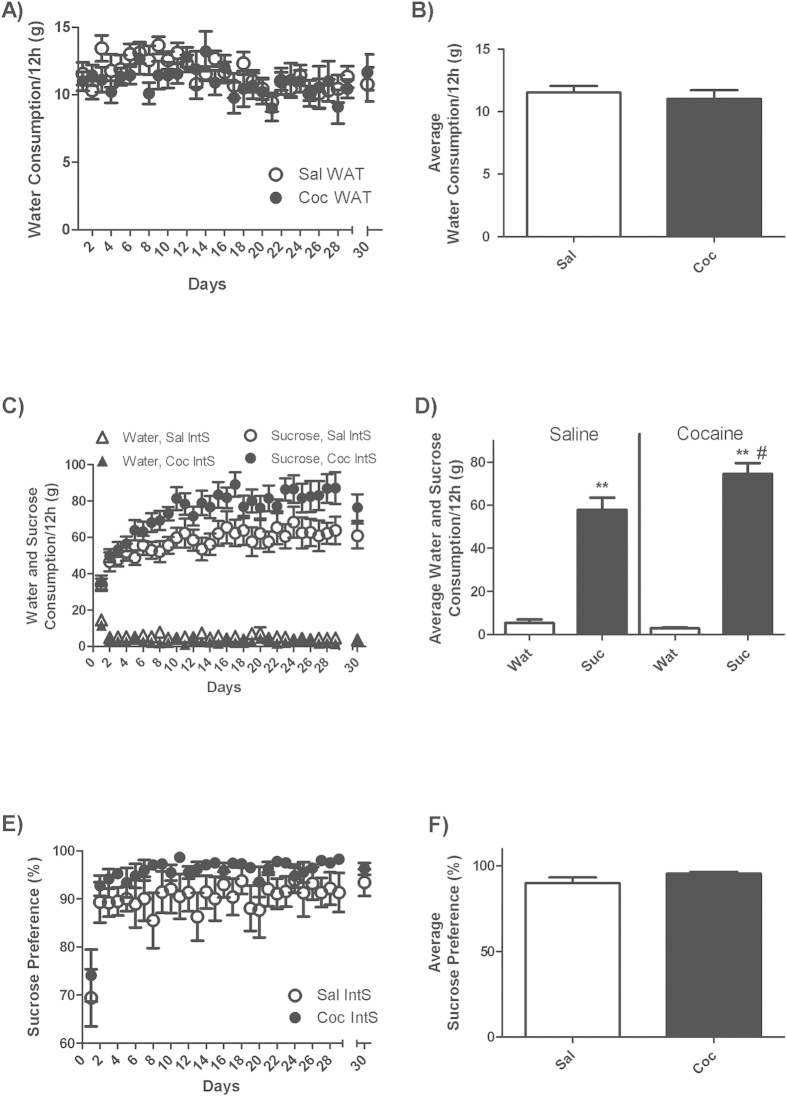
Water (Wat) and sucrose (Suc) consumption in rats with intermittent access to sucrose. (**A**) Time-course of water consumption measured every day at the end of the 12 h access to food and (**B**) average water consumption during withdrawal period for yoked-saline (Sal WAT) and cocaine-exposed (Coc WAT) groups in animals that had access to water only. (**C**) Time-course of sucrose and water consumption and (**E**) sucrose preference measured every day at the end of the 12 h access to food and sucrose and (**D**) average sucrose and water consumption and (**F**) average sucrose preference during the withdrawal period for yoked-saline (Sal IntS) and cocaine-exposed (Coc IntS) groups in animals that had intermittent access to sucrose. Notice that on the 30^th^ day of abstinence, liquid intake was measured during an 8 h instead of a 12 h period. Two-Way ANOVA followed by Student-Neuman-Keuls post-hoc test, **p < 0.01 Suc compared to WAT; #p < 0.05, Coc compared to Sal.

**Figure 5:**
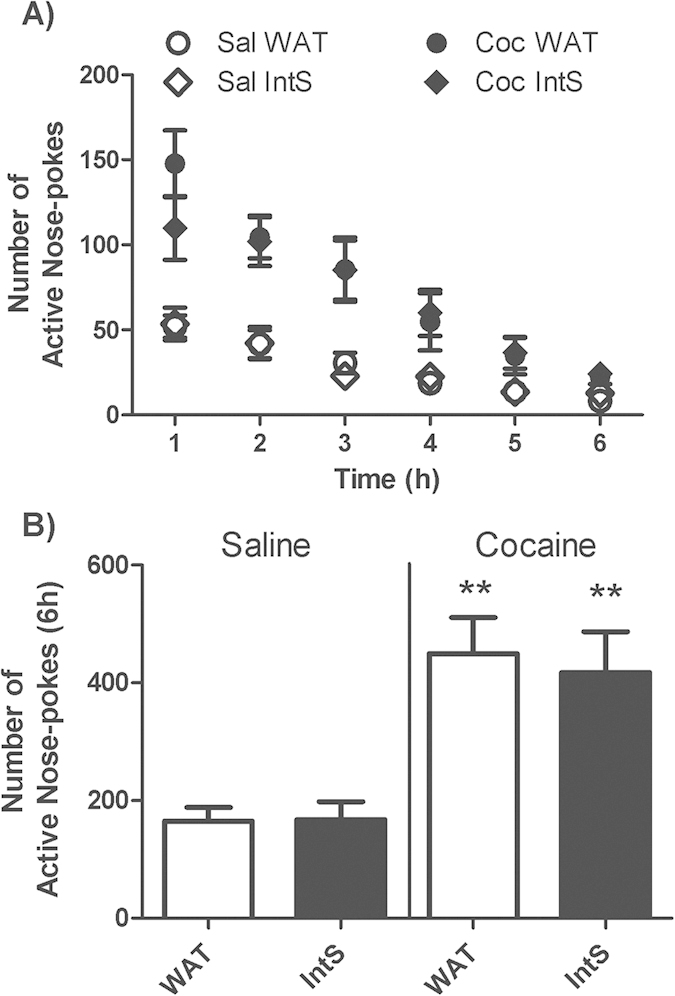
Lack of effect of intermittent exposure to sucrose during withdrawal period on cocaine seeking behavior. Time course (**A**) and total (**B**) cocaine seeking measured by the number of active nose-pokes during 6 h-session in yoked-saline and cocaine-exposed groups and access to water (WAT) or continuous sucrose (IntS) during a 30-day withdrawal period. Two-Way ANOVA followed by Student-Neuman-Keuls post-hoc test, **p < 0.01 cocaine compared to respective saline yoked controls.

Our general experimental design is schematized in Fig. 1. Half of the rats were allowed to self-administer cocaine, whereas the other half received saline infusions according to a yoked procedure. At the end of self-administration, rats were further divided pseudo-randomly into groups with different schedules of access to sucrose assuring similar levels of exposure to cocaine. For experiment 1, one group had a constant access to sucrose (10% w/v) and water (Continuous Sucrose, ContS), and the second group had access to water exclusively (Water, WAT). A total of 4 groups was obtained: 1) Coc ContS (n = 9); 2) Coc WAT (n = 9); 3) Sal ContS (n = 8); 4) Sal WAT (n = 9). For experiment 2, one group had access to sucrose (10% w/v) and water intermittently for 12 hours every 12 hours (Sucrose Intermittent, IntS) and one group had access to water exclusively (Water, WAT). A total of 4 groups was obtained: 1) Coc IntS (n = 9); 2) Coc WAT (n = 9); 3) Sal IntS (n = 9); 4) Sal WAT (n = 9). For each experiment, after a 30-day period of withdrawal in these different conditions of sucrose access, rats were tested for cocaine-seeking behavior.

The 10% (w/v) sucrose concentration was chosen because it represented a good compromise for the different aspects addressed in this study. In fact, rats vigorously self-administer solutions at this concentration^47^, they show a strong preference for this concentration in a choice procedures^48^ and they develop binge-like behavior when exposed intermittently to this concentration^27^.

### Self-administration apparatus and procedure

For cocaine self-administration experiments, we used experimental chambers equipped with nose-pokes as operanda and controlled by Imetronic interfaces and software (Imetronic, Pessac, France; www.imetronic.com). Rats were allowed to self-administer cocaine for ten experimental sessions that lasted 6 h each, using a Fixed Ratio 1 (FR1) schedule of reinforcement as previously described^2^, a regimen of exposure that has been associated with escalation of drug taking and development of addiction-like behaviors^49^. During daily sessions, a single response in the active nose-poke hole immediately delivered an i.v. injection of cocaine (0.15 mg/injection) and causes the house light to pulse for 5 s followed by a 5-s time-out. During this 5-s time-out period, the chambers were dark and responding had no programmed consequences. Responses in the inactive nose-poke hole were recorded but had no programmed consequences. Control “yoked” rats received an injection of saline, each time the paired “master” rat self-administered an injection of cocaine. Responses in the active and inactive nose-poke hole by “yoked” rats were recorded but had no programmed consequences.

### Withdrawal and exposure to sucrose

At the end of the last self-administration session, rats were pseudo-randomly divided in groups with different schedules of sucrose access assuring that the levels of cocaine self-administration were similar in all groups. Water and sucrose consumption was measured in grams by the difference between consecutive weights, for each experiment the side (left and right) was switched at each weighing to avoid development of a side preference.

For experiment 1, during a 30-day withdrawal period, in their home cage, rats had access to water only (WAT) or water and sucrose continuously (ContS). For the WAT groups, rats had access to two bottles of water (WAT). For the ContS groups, rats had continuous access to one bottle of water and one bottle containing sucrose (10%w/v). Bottles were weighted every 48 h. This group was designed to allow continuous exposure to a non-drug reward consistent with the continuous exposure to EE that we used in previous studies^1^,^2^.

For experiment 2, rats were maintained on a 12-hr deprivation period (no food, only water), followed by 12-hr access to one bottle of sucrose (10% w/v), one bottle of water (IntS) and food ad libitum according to the protocol developed by Hoebel and colleagues^27^. Control groups had access to water only (WAT). The feeding and sucrose schedule access started 4-hr into the dark cycle. Water and sucrose bottles were weighed at the end of the 12-hr period. This feeding schedule induces binge-like consumption, behavioral and neurochemical signs of sucrose addiction such as symptom of withdrawal, increases intake after abstinence and increases in dopamine in the nucleus accumbens following repeated binging^27^. Liquid consumption was calculated over 12-hr period of access. On the 30^th^ day of abstinence, exposure to sucrose lasted 8 instead of 12 h in order to perform cocaine-seeking test at the same time as self-administration sessions (8 AM).

### Test for cocaine seeking behavior

After 30 days of withdrawal, rats were brought to the self-administration room and tested for cocaine seeking. For all rats that had access to sucrose, sucrose was accessible until the beginning of the test in order to avoid possible withdrawal effects. This choice is consistent with our previous experiments with environmental enrichment in which rats were kept in their environment until the beginning of the test^1^,^2^. During these test sessions, responses in the active nose-poke hole produced the same stimuli (light and noise of the pump) produced during cocaine self-administration sessions, but the syringes were removed from the injection pumps and therefore cocaine was not delivered. Responses in the inactive holes were counted but had no consequence. The 6 h extinction session was divided into six 1-h extinction segments, separated by 5 min intervals. The number of active nose-pokes was used as a measure of cocaine seeking.

### Drugs

Cocaine HCl was obtained from Cooper (France) and dissolved in sterile saline (0.9% NaCl).

### Statistical Analysis

Differences in sucrose consumption and drug-seeking behavior were assessed by two-way ANOVA for repeated measures. For sucrose consumption, we used time (days of abstinence and exposure to sucrose) and drug (cocaine or saline) as factors. For experiment 2, since on the 30^th^ day, sucrose availability was limited to 8 h, only days 1–29 were used for statistical analysis. For cocaine seeking, we used drug (cocaine or saline), and schedule of access (WAT, ContS or IntS) as factors. Results showing significant overall changes were subjected to Student–Newman–Keuls post hoc test. Differences were considered significant when p < 0.05.

## Additional Information

**How to cite this article**: Nicolas, C. *et al.* Exposure to sucrose during periods of withdrawal does not reduce cocaine-seeking behavior in rats. *Sci. Rep.* **6**, 23272; doi: 10.1038/srep23272 (2016).

## Supplementary Material

Supplementary Figure legends
