# Supplementary material for: Exposure to sucrose during periods of withdrawal does not reduce cocaine-seeking behavior in rats
Source: Sci Rep. 2016 Mar 21;6:23272. doi: 10.1038/srep23272 (PMC4800416; doi:10.1038/srep23272)
Supplement: Supplementary Figure legends [file srep23272-s1.pdf]

Exposure to sucrose during periods of withdrawal does not reduce cocaine-seeking behavior in rats

Céline Nicolas, Claire Lafay-Chebassier & Marcello Solinas

Supplementary Figures and Legends.

Fig.S1

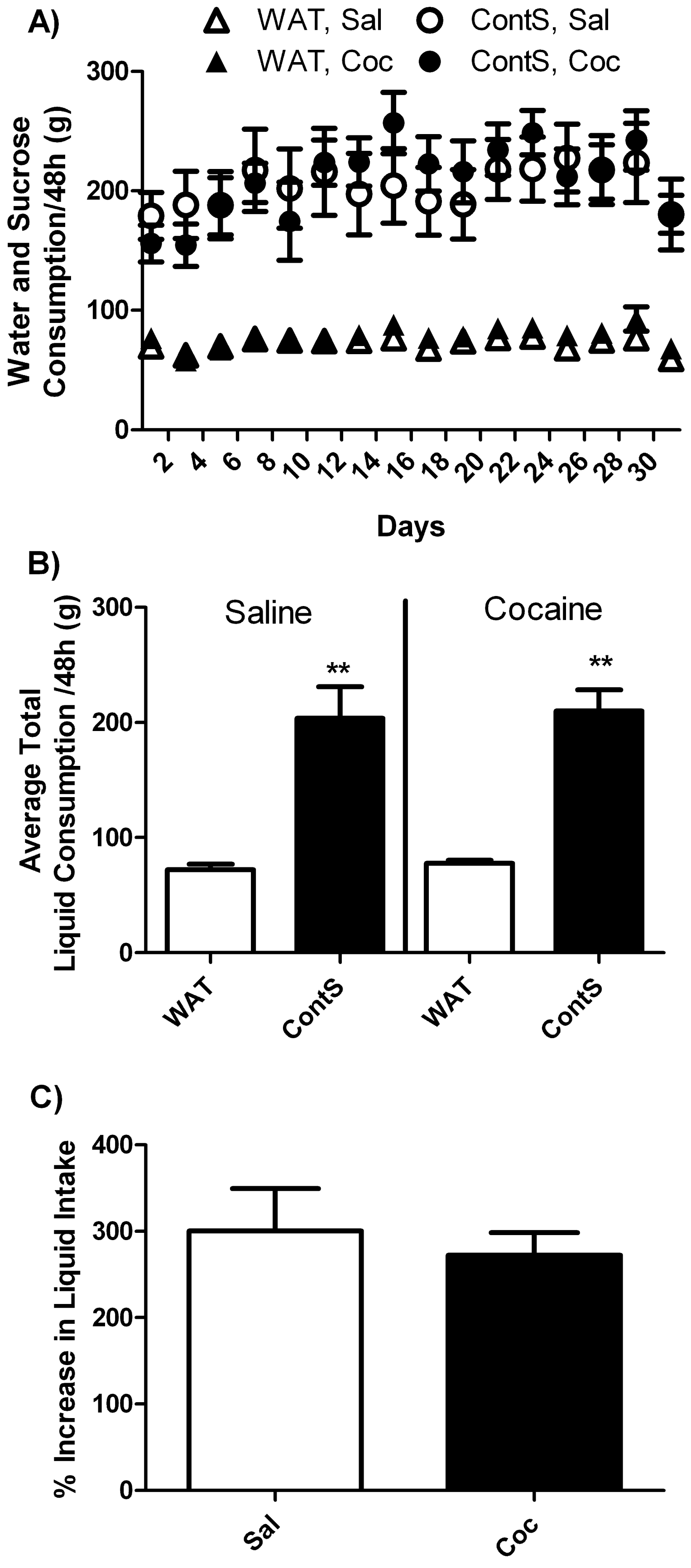
A)
WAT, Sal
ContS, Sal
WAT, Coc
ContS, Coc
Water and Sucrose Consumption/48h (g)
0
100
200
300
2
4
6
8
10
12
14
16
18
20
22
24
26
28
30
Days
B)
Average Total Liquid Consumption /48h (g)
0
100
200
300
Saline
Cocaine
**
**
WAT
ContS
WAT
ContS
C)
% Increase in Liquid Intake
0
100
200
300
400
Sal
Coc

Fig. S1. Comparison of total liquid consumption between animals with access to Water only (WAT) or to sucrose and water continuously (CoS). A) Time course of total liquid consumption and B) average total liquid consumption at 48h intervals during the 30-days of withdrawal period in rats with a history of cocaine self-administration and saline-yoked controls with continuous access to water only or water and sucrose. C) Increase in liquid consumption in Sal- and Coc-exposed rats with access to water only or sucrose and water consciously. Two-Way ANOVA followed by Student-Neuman-Keuls post-hoc test, ** < 0.01 ContS compared to WAT.

Fig.S2

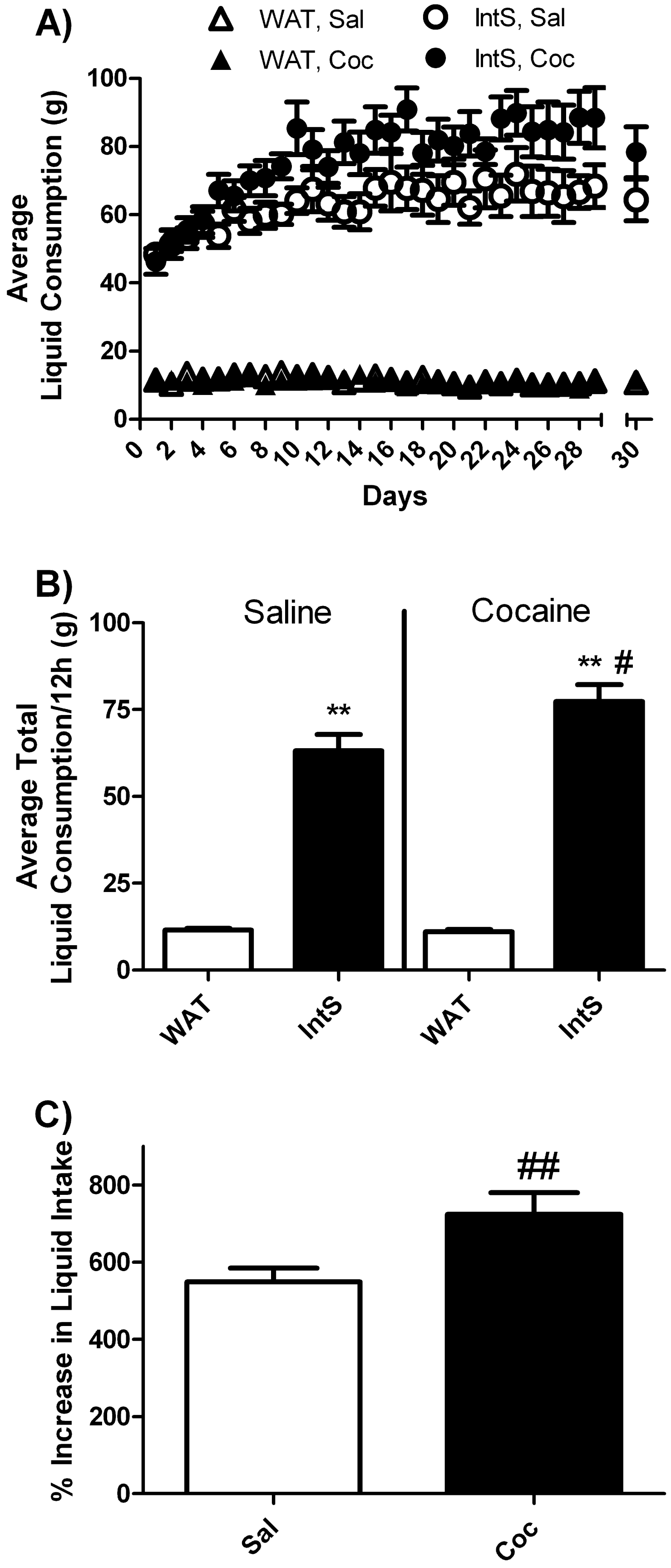
A)
WAT, Sal
WAT, Coc
IntS, Sal
IntS, Coc
Average Liquid Consumption (g)
100
80
60
40
20
0
0 2 4 6 8 10 12 14 16 18 20 22 24 26 28 30
Days
B)
Saline
Cocaine
Average Total Liquid Consumption/12h (g)
100
75
50
25
0
**
** #
WAT
IntS
WAT
IntS
C)
% Increase in Liquid Intake
800
600
400
200
0
##
Sal
Coc

Fig. S2. Comparison of total liquid consumption between animals with access to water only (WAT) or to sucrose and water intermittently (IntS). A) Time course of total liquid consumption and B) average total liquid consumption during the 12h of access to food during the 30-days of withdrawal period in rats with a history of cocaine self-administration and saline-yoked controls with intermittent access to water only or water and sucrose. C) Increase in liquid consumption in Sal- and Coc-exposed rats with access to water only or sucrose and water intermittently. Two-Way ANOVA followed by Student-Neuman-Keuls post-hoc test, ** < 0.01 IntS compared to WAT; # and ##, $p < 0.05$ and 0.01, cocaine compared to respective saline yoked control.
